# Necroptosis in Pneumonia: Therapeutic Strategies and Future Perspectives

**DOI:** 10.3390/v16010094

**Published:** 2024-01-07

**Authors:** Xiuzhen Mei, Yuchen Zhang, Shu Wang, Hui Wang, Rong Chen, Ke Ma, Yue Yang, Ping Jiang, Zhixin Feng, Chao Zhang, Zhenzhen Zhang

**Affiliations:** 1 College of Veterinary Medicine, Nanjing Agricultural University, Nanjing 210095, China; 2 Institute of Veterinary Medicine, Jiangsu Academy of Agricultural Sciences, Nanjing 210014, China; 3 GuoTai (Taizhou) Center of Technology Innovation for Veterinary Biologicals, Taizhou 225300, China; 4 Jiangsu Key Laboratory for Aquatic Crustacean Diseases, College of Marine Science and Engineering, Nanjing Normal University, Nanjing 210023, China; 5 College of Life Sciences, Nanjing Agricultural University, Nanjing 210095, China; 6 School of Traditional Chinese Pharmacy, China Pharmaceutical University, Nanjing 210009, China

**Keywords:** pneumonia, necroptosis, drug classification, programmed cell death

## Abstract

Pneumonia remains a major global health challenge, necessitating the development of effective therapeutic approaches. Recently, necroptosis, a regulated form of cell death, has garnered attention in the fields of pharmacology and immunology for its role in the pathogenesis of pneumonia. Characterized by cell death and inflammatory responses, necroptosis is a key mechanism contributing to tissue damage and immune dysregulation in various diseases, including pneumonia. This review comprehensively analyzes the role of necroptosis in pneumonia and explores potential pharmacological interventions targeting this cell death pathway. Moreover, we highlight the intricate interplay between necroptosis and immune responses in pneumonia, revealing a bidirectional relationship between necrotic cell death and inflammatory signaling. Importantly, we assess current therapeutic strategies modulating necroptosis, encompassing synthetic inhibitors, natural products, and other drugs targeting key components of the programmed necrosis pathway. The article also discusses challenges and future directions in targeting programmed necrosis for pneumonia treatment, proposing novel therapeutic strategies that combine antibiotics with necroptosis inhibitors. This review underscores the importance of understanding necroptosis in pneumonia and highlights the potential of pharmacological interventions to mitigate tissue damage and restore immune homeostasis in this devastating respiratory infection.

## 1. Introduction

Pneumonia, a severe pulmonary complication instigated by an array of factors including bacteria, viruses, and smoke, often leads to respiratory failure and mortality, representing a substantial clinical and economic burden as well as a significant public health challenge [1,2]. A number of epidemiological studies have reported sex differences in the incidence of pneumonia, which is higher in men and increases with age in both sexes [3,4,5,6]. It is well known that pathogens typically undergo a process from colonization to infection, and that many host-related factors, for example smoke exposure and chronic obstructive pulmonary disease, facilitate this process [7,8]. In vitro analysis of human peripheral cells has shown that, like murine macrophages, human male neutrophils express higher levels of toll-like receptor (TLR)4 and release more tumor necrosis factor (TNF)-α when exposed to lipopolysaccharide (LPS) than female neutrophils [9]. The therapeutic landscape of pneumonia has been dominated by the use of antibiotics for approximately 77 years, with β-lactam antibiotics being predominantly employed [10]. The emergence of bacterial resistance to β-lactams has necessitated the development of novel antibiotics such as penicillins, cephalosporins, and carbapenems. However, the efficacy of antibiotic therapy is hindered by regional variations in appropriate dosing and administration methods, coupled with diverse resistance patterns of bacterial strains in different locales [11].

The World Health Organization highlights that in developing countries, only 70% of pneumonia patients receive adequate antibiotic treatment, with patient compliance hovering around 50%. Furthermore, less than half of the nations have implemented effective strategies to enhance the quality of antibiotic use [12]. Notably, there exists a contentious debate regarding the modality of antibiotic administration, with several studies advocating intravenous over oral antibiotics [13,14,15], while others support oral antibiotic therapy [16,17]. Non-antibiotic therapies for pneumonia have also garnered significant research interest. For instance, a case study on phage therapy as an adjunct to intravenous antibiotics in treating recalcitrant pneumonia showed good tolerance to phage therapy without adverse events during or post-treatment [18]. Nevertheless, challenges such as the limited availability of phage and susceptibility testing facilities impede clinical trials [19].

In the pathogenesis of pneumonia, the role of necroptosis, a regulated form of necrotic cell death, is increasingly recognized. Pathogens counteract or evade the host immune system by secreting various virulence factors [20]. Necroptosis plays a pivotal role in the pathogenicity of these microorganisms and the etiology of pneumonia. For example, during bacterial pneumonia, most pathogens release cytotoxic products, with pore-forming toxins (PFTs) being a common cytotoxic product, capable of annihilating respiratory cells [21,22,23]. The dynamic equilibrium between cell death, proliferation, and differentiation is crucial for the development and homeostasis of multicellular organisms. Cell death serves as a critical function in growth, development, homeostasis, inflammation, immunity, and various pathophysiological processes [24,25]. Traditionally, cell death is categorized into three fundamental types based on morphological characteristics, modality, and regulatory control: (1) Type I cell death or apoptosis; (2) Type II cell death or autophagy; and (3) Type III cell death or necrosis [24,25]. Long perceived as a rapid, unregulated, passive process induced by pathological stimuli, the understanding of necrosis has evolved. Researchers now recognize necrosis as not purely unregulated cell death. In organisms, necrosis in certain cells is triggered by specific signals and undergoes a rigorously programmed death process, known as programmed necrosis or necroptosis [26,27].

Necroptosis is implicated in various diseases, including cancer [28], neurodegenerative diseases [29], and acute kidney injury [30], where its overactivation exacerbates pulmonary tissue inflammation. The use of specific compounds to prevent the overactivation of necroptosis has shown efficacy in halting disease progression [31,32]. These findings suggest that targeting necroptosis inhibition could offer a potential therapeutic strategy for pneumonia. Herein, we review the role of necroptosis in the pathogenesis of pneumonia induced by various pathogens. Importantly, we summarize several current necroptosis inhibitors and discuss the potential of targeting necroptosis as a therapeutic strategy for pneumonia.

## 2. Necroptosis in Pneumonia: Implications for Pathogenesis and Immune Response

**Figure 1:**
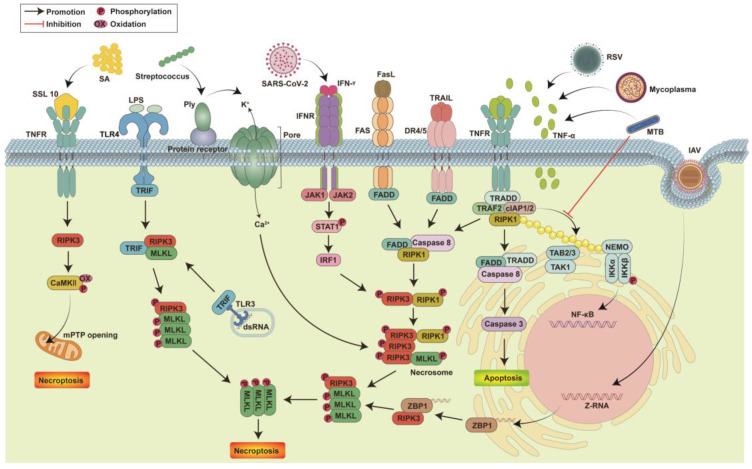
The mechanism by which pathogenic infection causes cell necroptosis. Pathogens trigger the necroptosis signaling pathway by activating multiple death receptors on the cell surface. The binding of TNF-α to TNFR triggers three different signaling responses: NF-κB activation, apoptosis or necroptosis. Respiratory syncytial virus (RSV), Mycoplasma hyopneumoniae and Mycobacterium tuberculosis (MTB) can induce cells to secrete TNF-α, thereby inducing the formation of RIPK1-RIPK3-MLKL necrosomes in cells and triggering necroptosis. MTB also prevents NF-κB activation by inhibiting RIPK1 ubiquitination. Death ligands, including FasL and TRAIL, trigger necroptosis by inducing necrosome formation by binding to the corresponding death receptors. SARS-CoV-2 activates IFNR by secreting IFN-γ and triggers necrosome formation through JAK-STAT1 to induce cell necroptosis. Streptococcus pneumoniae releases the pore-forming toxin pneumolysin (Ply) to induce cell damage and ion dysregulation, thereby triggering the formation of necrosomes in a death receptor-independent manner. Lipopolysaccharide (LPS) and double-stranded RNA (dsRNA) activate necroptosis through TRIF-mediated necrosome complex formation. SSL10 secreted by Staphylococcus aureus (SA) binds to TNFR and induces cell necroptosis by activating the RIPK1-RIPK3-MLKL and RIPK3-CaMKII mitochondrial permeability transition pore pathways. The Z-RNA produced by intracellular replication of Influenza Avirus (IAV) binds to ZBP1, leading to RIPK1-independent necroptosis through the ZBP1-RIPK3 complex.

Necroptosis is a strictly regulated form of cell death, sharing morphological characteristics with apoptosis and necrosis, primarily presenting as cellular swelling and plasma membrane rupture [33,34]. This form of non-caspase-dependent cell death critically depends on the kinase activities of receptor-interacting serine/threonine-protein kinase 1 (RIPK1) (in some contexts), receptor-interacting serine/threonine-protein kinase 3 (RIPK3), and mixed-lineage kinase domain-like protein (MLKL), triggered by death receptors, including pathogen recognition receptors and tumor necrosis factor receptor 1 (TNFR1). The interaction and phosphorylation of RIPK1 and RIPK3 through their RIP homotypic interaction motif (RHIM) domains lead to the formation of necrosomes, central to necroptosis signaling [35,36]. Active RIPK3 phosphorylates MLKL, causing its oligomerization and membrane translocation, leading to membrane rupture and release of intracellular contents acting as alarmins [37,38,39,40]. Necroptosis releases damage-associated molecular patterns (DAMPs) and cytokines, inducing inflammatory responses and exacerbating tissue damage [41,42]. Pneumonia, caused by various pathogens, including bacteria and viruses, is characterized by cytokine dysregulation and exacerbated inflammatory responses due to leukocyte infiltration [43,44,45]. Increasing evidence suggests necroptosis plays a significant role in pneumonia pathogenesis. The necroptotic death of immune cells, including macrophages and neutrophils, impacts immune responses during pneumonia. Besides RIPK1, RIPK3 activation can also be mediated by other RHIM-domain-containing proteins, such as Z-DNA-binding protein 1 (ZBP1, also known as DAI/DLM-1) and Toll/IL-1 receptor (TIR) domain-containing adapter protein inducing interferon (IFN)-β (TRIF, also known as TICAM-1) [46,47,48,49]. Necroptosis also involves the activation of Ca^2+^/calmodulin-dependent protein kinase (CaMKII) in response to reactive oxygen species (ROS)-mediated damage and intracellular Ca^2+^ changes [50,51]. Necroptosis can be induced by several ligand-receptor binding signaling pathways, including TNF-α/TNFR1, Fas-ligand (FasL)/Fas, interferon (IFN)-γ/interferon-α/β receptor subunit 1 (IFNAR1), double-stranded RNA/TLR3, and double-stranded DNA/ZBP1, converging on RIPK3 to execute necroptosis (Figure 1).

### 2.1. Viral-Induced Necroptotic Cell Death in Pneumonia

Necroptosis, an immunogenic cell death pathway, eliminates virus-infected cells and mobilizes innate and adaptive immune responses to limit viral replication [52]. However, the benefits of necroptosis for the host may sometimes be offset by potentially harmful excessive inflammatory consequences [53]. Necroptosis in viral infections can be a double-edged sword [54]. It can lead to cell suicide, thereby preventing viral replication and further disease progression [42]. When apoptosis is inhibited, necroptosis stimulates robust antiviral immune responses and accelerates viral clearance from infected organs [55]. However, as cells rupture, intracellular viruses can evade cell death and spread throughout the body [56]. Uncontrolled lytic cell death can lead to tissue damage and severe diseases, including acute respiratory distress syndrome (ARDS), neurodegenerative diseases, and inflammatory disorders [56].

Necroptosis creates a rich inflammatory environment by releasing DAMPs and recruiting immune cells and chemotactic or cytokine factors to prevent viral infections [57]. The COVID-19 pandemic caused by SARS-CoV-2 has seen an excessive immune response and cytokine production at infection sites [58]. Key cytokines identified in SARS-CoV-2 pathology include TNF-α, interleukin (IL)-1β, and IL-6, commonly released during necroptosis [59,60]. SARS-CoV-2 infection activates multiple cell death pathways in lung epithelial cells, including necroptosis, apoptosis, and pyroptosis, leading to an irreversible cytokine storm and multiorgan failure [61,62]. SARS-CoV-2 infection of lung epithelial cells induces caspase-8 activation, triggering apoptosis and processing inflammatory cytokines into bioactive forms, including IL-1β. IL-1β secretion via the SARS-CoV-2-induced necroptosis pathway leads to an inflammatory response [61]. A recent study reported that TNF-α and IFN-γ synergistically drive cytokine storm and cell death associated with COVID-19 and sepsis. The combined action of TNF-α and IFN-γ induces RIPK1 kinase activity-dependent cell death (including apoptosis, pyroptosis, and necroptosis) through the JAK-STAT1-IRF1 axis [62].

Influenza virus (IAV) infection can induce severe pneumonia, where a pro-inflammatory cytokine storm is a significant hallmark and closely associated with lung/respiratory dysfunction [63]. Research has indicated that IAV-induced necroptosis is crucial for host antiviral defense and is associated with inflammatory responses [55,64]. Studies have found that IAV triggers RIPK3-dependent death in infected cells. Host protein ZBP1 is required for RIPK3 activation and cell death in mouse cells infected with IAV. ZBP1, containing the RHIM domain, recruits RIPK3 through RHIM-RHIM interactions, then promotes MLKL-dependent and RIPK1-independent necroptosis [65,66,67]. ZBP1’s Zα domains specifically bind Z-DNA and Z-RNA [68,69,70]. ZBP1 recognizes Z-RNA produced by the replication of the IAV genome through its second Zα domain, binds RIPK3 to activate MLKL, and triggers necroptosis [66,71,72]. Gaba et al. found that IAV’s NS1 protein participates in necroptosis by interacting with MLKL [73]. This interaction leads to increased MLKL oligomerization and membrane translocation, subsequently activating the Nod-like receptor protein 3 (NLRP3) inflammasome and releasing IL-1β, thereby enhancing antiviral defense and leading to viral clearance. Additionally, macrophage death in IAV infection is associated with excessive production of local and systemic cytokines/chemokines, cellular infiltration, extracellular matrix degradation, and airway epithelial shedding [74]. Macrophages play a crucial role in clearing viral particles and initiating adaptive immune responses [75]. A recent study found that macrophages exposed to IAV undergo inflammatory programmed cell death, leading to a cycle involving TLR4, TNF-α, and RIPK1, resulting in an imbalance of pro-inflammatory and regulatory mediators associated with extracellular and in vivo cytokine storms and severe influenza [76]. Therefore, necroptosis may be a double-edged sword in IAV infection. While effectively eliminating viral replication, abnormal or uncontrolled cell death can lead to pulmonary dysfunction and inflammation, resulting in morbidity and mortality, depending on the severity of infection and host cell response [77,78].

Respiratory syncytial virus (RSV) is a common pathogen worldwide causing respiratory infections [79]. It can cause varying degrees of pulmonary inflammation in children [80,81]. Simpson et al.’s study first reported that RSV infection induces necroptotic cell death in airway epithelial cells (AECs) [82]. Inhibiting RIPK1 or MLKL weakens RSV-induced high mobility group box 1 (HMGB1) translocation and release, reducing viral load. Additionally, another study found that during RSV infection of alveolar macrophages (AMs), AMs secrete TNF-α, which triggers macrophage necroptotic death in a RIPK1, RIPK3, and MLKL-dependent manner. The necroptosis pathway is detrimental to alveolar macrophage clearance of RSV, further exacerbating lung injury [83].

### 2.2. Bacterial-Induced Necroptotic Cell Death in Pneumonia

In addition to viral-induced pneumonia, cellular necrosis and tissue damage caused by bacterial infections also lead to significant pulmonary pathology. During Mycobacterium tuberculosis (MTB) infection, the pathogen invades host alveolar macrophages, releasing inflammatory cytokines such as TNF-α, resulting in intense local inflammatory responses [84]. A complex relationship exists between MTB, TNF-α, and necroptosis during infection. Studies have shown that MTB infection triggers RIPK1/RIPK3/MLKL-dependent necroptotic signaling pathways due to excessive TNF-α release [85]. RIPK3 plays a dual role in MTB infection: inducing mitochondrial reactive oxygen species (mROS) production to kill bacteria and mediating macrophage necroptosis, thereby releasing MTB extracellularly and promoting bacterial growth [86]. MTB infection reshapes intracellular signaling by upregulating MLKL, TNFR1, and ZBP1 in macrophages, while downregulating cellular inhibitor of apoptosis 1 (cIAP1), creating a pro-necroptotic environment [87]. Necroptotic cell death requires limited caspase 8 activity but not its elimination, as caspase 8 auto-cleavage and dimerization upon TNF-α receptor binding inactivates RIPK1 and RIPK3, inducing apoptosis. In MTB-infected macrophages, caspase 8 levels remain unchanged, but cellular FLICE-like inhibitory protein (cFLIP) increases. cFLIP forms heterodimers with un-cleaved caspase 8, preventing caspase 8 homodimer formation and activation, thus inhibiting apoptosis [87,88]. These caspase 8/cFLIP heterodimers also possess partial catalytic function to cleave RIPK1, RIPK3, and cylindromatosis lysine 63 deubiquitinase (CYLD), simultaneously inhibiting MLKL and necroptotic pathway activation [89,90,91,92]. MTB infection also significantly reduces CYLD in macrophages, a key enzyme for removing certain ubiquitin chains from RIPK1 in necrosomes [93]. CYLD removal is necessary for RIPK1 and RIPK3 phosphorylation and function, hence its loss can protect cells from necroptosis [93,94]. The ratio between cFLIP and caspase 8 is a key determinant of cell fate [95]. It is suggested that increased cFLIP may promote caspase 8-mediated degradation of CYLD and other necrosomal components, potentially abrogating MTB-induced necroptotic signaling [96].

Streptococcus pneumoniae, a common pathogen in community-acquired pneumonia, induces RIPK3-MLKL-dependent necroptosis in the lungs [97,98]. RIPK3-initiated necroptosis is crucial for host defense against Streptococcus pneumoniae, forming complexes with RIPK1, MLKL, and MCU (mitochondrial calcium uniporter) during infection to induce mitochondrial calcium uptake and mROS production. In macrophages, RIPK3 initiates necroptosis through mROS-mediated mitochondrial permeability transition pore opening and activates NLRP3 inflammasomes via the mROS-AKT pathway to prevent Streptococcus pneumoniae [99]. RIPK3 regulates the balance between pro-inflammatory signals for bacterial clearance and fatal consequences of excessive inflammation. While RIPK3 initiates NLRP3 inflammasome activation, secreting pro-inflammatory cytokines, guiding immune cell recruitment and bacterial clearance, it also modulates necroptosis to clear dying bacteria and cell debris, preventing excessive inflammation and maintaining immune homeostasis. During bacterial pneumonia, most pathogens release cytotoxic products that kill respiratory cells, primarily pore-forming toxins (PFTs), leading to ionic dysregulation and necroptosis activation [23,100]. Streptococcus pneumoniae PFTs, particularly pneumolysin (Ply), lead to the imbalance of Ca^2+^ and K^+^ in cells after cell membrane injury, which can then activate RIPK1, RIPK3 and MLKL in a death receptor-independent manner, inducing necroptotic cell death in lung epithelial cells [100].

Staphylococcus aureus (SA) infection causes highly inflammatory pneumonia. Toxins secreted by SA, targeting specific receptors on human and mouse cells, induce necroptotic cell death in host immune cells, contributing significantly to SA pneumonia pathology. SA toxins induce RIPK1/RIPK3/MLKL-dependent necroptosis in lung macrophages and the production of pro-inflammatory cytokines TNF-α, IL-6, IL-1α, and IL-1β [101]. Alpha-hemolysin (Hla) recognizes ADAM-10 in the lungs and activates the NLRP3 inflammasome, stimulating IL-1β and IL-18 production [102,103]. Phenol-soluble modulins (PSMs), closely associated with SA pathogenicity, with alpha-type PSMs (PSMα) responsible for significant in vivo leukocidal activity, reportedly induce TNF-α secretion, triggering MLKL-mediated neutrophil necroptosis [104,105,106]. A recent study revealed that a staphylococcal superantigen-like family protein, SSL10, produced by SA, triggers endothelial and epithelial cell necroptosis by binding to TNFR1 on cell membranes. Upon binding, two different downstream pathways of TNFR1 are activated in a RIPK3-dependent manner: the RIPK1-RIPK3-MLKL and RIPK3-CaMKII mitochondrial permeability transition pore pathways [107].

Mycoplasma is a common respiratory pathogen causing pneumonia in humans and animals, which leads to primary atypical pneumonia with notable lung inflammation and immune dysfunction in severe infections [108]. Bacterial cellular components, metabolites and toxins released from Mycoplasma pneumoniae can induce immune-pathological damage in the host tissues. The Ca^2+^-dependent cytotoxic nuclease (encoded by MPN133) produced by Mycoplasma pneumoniae can lead to apoptotic-like programmed cell death in the host. Mycoplasma pneumoniae lipoproteins can be recognized by TLRs, which stimulate the release of pro-inflammatory cytokines including TNF-α, IL-1β, and IL-6 among others [109,110]. Mycoplasma gallisepticum infection causes severe inflammation of the respiratory tract in chickens [111]. One study found that phenylalanine increased RIPK1 and RIPK3 by increasing the expression level of gga-miR-190a-3p, aggravating necroptosis of infected cells, thereby leading to inflammatory damage caused by Mycoplasma gallisepticum infection [112]. Mycoplasma hyopneumoniae infection induces high levels of TNF-α production in alveolar macrophages, with excessive TNF-α secretion activating the necroptotic signaling pathway through RIPK1, RIPK3, and MLKL-dependent mechanisms, causing lung inflammation [113].

### 2.3. Necroptotic Cell Death in Pneumonia under Non-Infectious Conditions

In addition to pathogen-triggered necroptosis leading to pulmonary inflammation, various non-pathogenic factors inducing pneumonia can also initiate necroptotic cell death. Unlike pathogens that secrete ligands or interact directly with corresponding receptors on cells to activate necroptotic signaling, sterile injuries cause direct induction of cellular reactive oxygen species (ROS) or release of high mobility group box 1 (HMGB1), subsequently triggering intracellular signal transduction and release of various molecules and cytokines [114].

Acute lung injury/acute respiratory distress syndrome (ALI/ARDS) represents a common and fatal complication in critical illnesses, characterized by increased pulmonary microvascular endothelial cell permeability, destruction of alveolar epithelial tight junctions, and inflammatory damage [115,116]. ARDS is triggered by various etiologies, with pneumonia being the most common cause, followed by extrapulmonary sepsis, aspiration, non-cardiogenic shock, transfusion, and trauma [117,118]. The pathogenesis of ARDS involves inflammatory cell accumulation, oxidative stress, and cellular death [116]. In an oleic acid (OA)-induced ARDS rat model, the RIPK1/RIPK3/MLKL-dependent necroptotic pathway was activated, leading to neutrophil infiltration and protein leakage into the lung tissues [119]. Increasing research has identified intricate crosstalk among different cell death pathways in the pathogenesis of ARDS, where interactions between necroptosis, ferroptosis, and pyroptosis can synergistically lead to cell death [120]. Moreover, red blood cell transfusion, through the release of HMGB1, enhances susceptibility to pulmonary inflammation and induces necroptosis in pulmonary endothelial cells, raising the risk of ARDS in transfusion patients [121].

Chronic obstructive pulmonary disease (COPD) is characterized by progressive airflow obstruction, emphysema, and abnormal pulmonary inflammation. An estimated 80% of cases are attributed to smoking, with other risk factors including biomass fuels, secondhand smoke, particulate matter (PM), dust, and toxic gases [122]. Studies have found that PM can induce RIPK and MLKL-dependent necroptotic cell death in human bronchial epithelial cells and mouse lungs, contributing to the pathogenesis of PM-induced lung damage [123]. Necroptosis has emerged as a potential mechanism in the pathogenesis of COPD. High concentrations of oxidants and reactive oxygen species (ROS) in cigarette smoke (CS) are key modulators in necroptotic signaling [124]. Exposure to CS has been shown to induce mitoROS production and activate the RIPK1/RIPK3/MLKL necroptotic pathway in alveolar macrophages and bone marrow-derived macrophages (BMDM), leading to pulmonary inflammatory responses and promoting COPD progression [125]. Chen et al. found that phosphorylated MLKL (p-MLKL) and HMGB1 release were significantly elevated in COPD patients, mice exposed to smoking, and lung epithelial cells treated with cigarette smoke extract (CSE) [126]. CSE-induced necroptotic cell death in lung epithelial cells released more DAMPs, leading to enhanced expression of pro-inflammatory cytokines TNF-α and IL-6 [126]. Mizumura et al. discovered that CS induces PINK1-dependent mitochondrial autophagy and dysfunction in epithelial cells, with increased expression of mitochondrial autophagy protein PINK1 and necroptotic regulator RIPK3 in lung tissues of COPD mice, suggesting that CS regulates cell death through initiation of mitochondrial autophagy and necroptosis, contributing to the pathogenesis of COPD [127].

## 3. Pharmacological Interventions against Necroptosis in Pneumonia

### 3.1. Synthetic Chemical Compounds

Necrostatin-1 (Nec-1) is the first identified RIPK1-specific inhibitor, extensively utilized as a tool compound in necroptosis mechanism studies [128,129]. Nec-1 effectively inhibits RIPK1 autophosphorylation, thereby reducing the interaction between RIPK1 and RIPK3, inhibiting the formation and/or stability of the RIPK1-RIPK3 complex, and impacting downstream RIP3 activity [128,130]. The protective effects of Nec-1 have been validated in numerous disease models [119,131]. Nec-1 protects mice from TNF-induced lethal systemic inflammatory response syndrome (SIRS) [132]. Pre-treatment with Nec-1 improved lung function in oleic acid (OA)-induced ARDS rats and significantly mitigated pulmonary edema [119]. Furthermore, Nec-1 reduces the RIPK1-RIPK3 interaction, downregulating the RIPK1-RIPK3-MLKL signaling pathway and inhibiting inflammatory responses by decreasing neutrophil infiltration and protein leakage into lung tissues in OA-induced ARDS [119]. Research indicates that Nec-1 significantly protects against inflammation and necroptosis in lipopolysaccharide-induced acute lung injury (ALI) [133]. Nec-1 reduces RIP1 and RIP3 expression in rat alveolar epithelial RLE-6TN cells, shielding the cells from swelling, nuclear fragmentation, and membrane rupture [133]. Pharmacokinetically, Nec-1 rapidly enters the bloodstream or circulatory system, reaching peak blood concentration within 1 h after oral administration in rats [115]. It is fully soluble in 95% ethanol, with an absolute bioavailability exceeding 50%. These characteristics endow Nec-1 with broad clinical application potential. However, Nec-1 has a short half-life, approximately 1–2 h in rats [115]. Additionally, Nec-1 has been described as an effective inhibitor of indoleamine 2,3-dioxygenase (IDO), a molecule with significant immunomodulatory activity [134]. IDO controls the flux between pathways leading to the production of pro-inflammatory or anti-inflammatory cytokines [134,135]. Thus, the application of Nec-1, while inhibiting necroptosis, also inhibits IDO. This off-target effect limits its specific clinical application targeting RIPK1 [129,136]. In contrast, its structural analog, 7-Cl-O-Nec-1 (Nec-1s), exhibits higher metabolic stability and dose-dependent inhibition of RIPK1, thus being more effective than Nec-1 and without any inhibitory effect on IDO [129,137]. Extensive research has demonstrated the therapeutic efficacy of Nec-1 in various disease models. Despite drawbacks such as metabolic instability and off-target effects, its protective role in disease models and potential for optimization cannot be overlooked. Structural optimization of Nec-1 could avoid its off-target effects, making Nec-1 and its analogs promising in the treatment of pneumonia.

Zhang and colleagues designed 23 benzothiazole derivatives targeting RIPK1, among which SZM-1209 showed high anti-necroptotic activity and RIPK1 kinase selectivity [138]. Pre-treatment with SZM-1209 in HT-29 cells inhibits the necroptotic signaling cascade of RIPK1/RIPK3/MLKL, thereby preventing the formation of necrosomes [138]. In a murine model of mTNF-α-induced systemic inflammatory response syndrome (SIRS), SZM-1209 completely reversed mortality with significant anti-inflammatory effects [138]. Moreover, it notably alleviates acute lung injury/acute respiratory distress syndrome (ALI/ARDS) by reducing lung edema and pathological damage [138]. The activity of SZM-1209 against RIPK1, necroptosis, SIRS, and ALI suggests the potential of optimized benzothiazoles as lead structures for treating ALI-related diseases.

Huang’s team identified a novel small-molecule salt-inducible kinase (SIK) inhibitor, HG-9-91-01, effectively inhibiting RIPK3 kinase activity to suppress necroptosis [139]. HG-9-91-01 blocks TNF-α or Toll-like receptor (TLRs)-mediated necroptosis, inhibiting RIPK3 autophosphorylation, its interaction with MLKL, and subsequent MLKL oligomerization. However, HG-9-91-01 also triggers RIPK1-RIPK3-caspase 1-caspase 8-dependent apoptosis, cleaving downstream GSDME to induce pyroptosis. Treatment with HG-9-91-01 protects mice from Staphylococcus aureus-mediated lung injury, highlighting SIKs inhibitor HG-9-91-01 as a novel RIPK3 kinase inhibitor and a potential therapeutic target for necroptosis-mediated inflammatory diseases [139].

Necrosulfonamide (NSA), identified through compound library screening, targets MLKL downstream of RIPK3 to block various stimuli-induced necroptosis [140]. However, NSA’s inhibition is species-specific, effective in human cells but not in murine cells, due to its targeting of MLKL’s Cys86 residue, which corresponds to a tryptophan residue in murine homologs [140]. NSA has shown efficacy in alleviating lung ischemia-reperfusion injury (IRI) in rats by inhibiting necroptosis [141,142]. However, NSA’s narrow SAR limits further development and restricts its use to in vitro studies, mainly in primate cells.

Cisatracurium besylate (CIS), a moderately potent non-depolarizing neuromuscular blocking agent, exhibits minimal periodic fluctuation and low histamine release. It is commonly used in clinical settings as an adjunct to general anesthesia or as a sedative in intensive care units [143]. Recent research has uncovered CIS’s dual role in inhibiting MTB and necroptotic cell death [144]. It exhibits high protective activity against MTB-infected macrophages by targeting necroptosis. CIS disrupts the interaction of RIPK3 with MLKL, inhibiting MLKL phosphorylation, oligomerization, and translocation to the cell membrane. It modulates RIPK3 autophosphorylation but does not interfere with the association between RIPK3 and its upstream kinases RIPK1 or ZBP1. It significantly inhibits Mycobacterium tuberculosis proliferation in human monocytic (THP-1), murine fibroblast (L929), mouse bone marrow-derived macrophages (BMDMs), and in mice, shows strong adjunct antibacterial effects. Its pharmacological advantages include strong and rapid effects, quick recovery, no accumulation, good controllability, and non-dependence on liver and kidney functions, leading to widespread clinical application [145,146]. However, some studies have indicated that CIS can affect cell proliferation [147]. The inhibitory effect of CIS on endothelial cells depends on drug concentration and treatment duration. High-dose and prolonged CIS treatment can damage endothelial cells, possibly due to excessive activation of mitochondrial fission and intracellular autophagy, leading to autophagic cell death [148]. Therefore, when considering CIS as a candidate compound for necroptosis, it is crucial to consider treatment duration and dosage to avoid adverse effects.

### 3.2. Natural Products as Interventions

**Figure 2:**
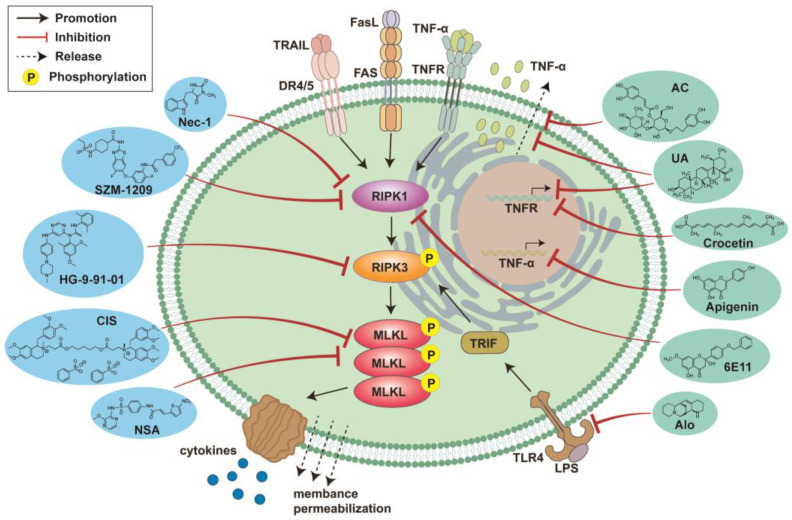
Pharmacological interventions against necroptosis in pneumonia. Cell necroptosis can be modulated by different drugs. Chemically synthesized drugs Necrostatin-1 (Nec-1), SZM-1209, HG-9-91-01, Cis-sulfonatequinacrine (CIS) and Necrosulfonamide (NSA) can inhibit RIPK1 or RIPK3 or MLKL in the necroptosis signaling pathway, thereby preventing cell necroptosis. A variety of natural products also have the effect of inhibiting cell necroptosis. Acteoside (AC) inhibits cell necroptosis by preventing the release of TNF-α. Ursolicacid (UA) can not only inhibit the production of TNFR at the transcriptional level, but also prevent the release of TNF-α, exerting a necroptosis inhibitory effect. Crocetin and apigenin inhibit cell necroptosis by inhibiting the transcription of TNFR and TNF-α respectively. 6E11 selectively inhibits RIPK1 thereby blocking the necroptosis pathway activated by TRAIL or TNF-α. Aloperine (Alo) inhibits cell necroptosis by reducing TLR4 levels and blocking the TRIF/RIPK3/MLKL signaling pathway.

Beyond the aforementioned synthetic compounds, certain natural product derivatives have been reported as RIPK1 inhibitors (Figure 2). 6E11, a flavonoid derivative isolated from the buds of black poplar, has been identified as a novel inhibitor of necroptosis. It exhibits its inhibitory action with an EC50 in the micromolar range and functions as a non-ATP competitive inhibitor, demonstrating significant selectivity for RIPK1 [149]. Studies have shown that 6E11 protects human bronchial organoids from necroptotic cell death [150]. 6E11 shows minimal or no cytotoxic effects on human peripheral blood leukocytes (PBL) or human retinal pigment epithelial cells (RPE-1 hTERT) within the concentration range of 1 to 25 µM [149]. When added to FADD-deficient Jurkat cells at a concentration of 10 μM, one to four hours after TNF treatment or necroptotic stimulation, 6E11 was able to reduce cell mortality from 60% to approximately 20% [149]. 6E11 protects human aortic endothelial cells (HAEC) from the effects of cold hypoxia-reoxygenation. Notably, the efficacy of 6E11 was observed to be significantly superior to necroptotic cell death control inhibitors (Nec-1 and Nec-1s) [149]. 6E11 inhibits TNF-α or TRAIL-induced necroptosis in a dose-dependent manner but does not inhibit TRAIL-induced apoptosis, providing a new structural type for RIPK1 inhibitors [149].

Ursolic acid (UA) and its isomer oleanolic acid (OLA), triterpenoid compounds found in various vegetables, fruits, and medicinal plants, are active components in antituberculosis herbal medicines, demonstrating low cytotoxicity and a broad range of pharmacological properties [151]. They inhibit bacterial load during MTB infection and show significant anti-mycobacterial activity [152,153,154,155]. UA’s efficacy varies across cell types; in MTB-induced mouse monocytic macrophages RAW 264.7 and type II alveolar cells A549, UA significantly reduces the transcriptional levels of TNF-α, IL-1β, and IL-6, and inhibits nitric oxide (NO) release [156]. It has been found that UA and OA can be preferentially recognized by different receptors on macrophages, CD36, and TGR5, respectively. Treatment with UA and OLA induces overexpression of CD36 and TGR5 receptors in J774 A.1 macrophages and stimulates the production of TNF-α, NO, and ROS while inhibiting TGF-β production, enabling the infected macrophages to control and eliminate intracellular mycobacteria [157]. Studies have found that UA can inhibit MTB-induced macrophage necroptosis and counteract the excessive inflammatory response caused by MTB-infected macrophages by regulating the host immune response [158]. Further mechanism studies have found that UA inhibits the release of TNF-α and overactivation of TNFR1 thus preventing the activation of the RIPK1-RIPK3-MLKL cascade and inhibiting cell necroptosis. UA also induces macrophage autophagy by inhibiting the Akt/mTOR pathway to enhance the intracellular killing of MTB [158]. In a study by Vinay et al., UA and OLA were co-delivered at a 1:1 ratio (each at a dose level of 120 μg/mL) to the lungs of rats using a metered dose inhaler (MDI) to assess their in vivo safety and pharmacokinetic parameters in a pulmonary tuberculosis rat model. The results showed that tracheal administration of UA and OLA via MDI is safe, with no toxic effects on animal organs and no adverse impact on overall animal health. Importantly, co-administration of UA and OLA resulted in an extended biological half-life, indicating prolonged circulation time in the body. Furthermore, the low peak plasma concentration was notable, suggesting higher lung drug concentration relative to systemic availability [159].

Apigenin, a pharmacologically active flavonoid compound found in various fruits, vegetables, and herbs, possesses multiple biological properties, including anti-inflammatory, antioxidative, anti-apoptotic, and antitumor effects [160,161,162,163,164]. Notably, apigenin significantly reduces IL-6 levels in lipopolysaccharide (LPS)-activated mouse macrophages and inhibits the production of cluster of differentiation 40 (CD40), TNF-α, and IL-6 by suppressing the phosphorylation of signal transducer and activator of transcription 1 (STAT1) in IFN-γ induced mouse microglia [165,166]. A recent study discovered that apigenin reverses TNF-α autocrine-induced necroptotic cell death in alveolar macrophages infected with Mycoplasma, alleviating lung damage caused by Mycoplasma infection [113]. Apigenin stimulates peroxisome proliferator-activated receptor γ (PPARγ) to promote the transcription of ubiquitin-like protein 1 with PHD and RING finger domains (Uhrf1), which blocks TNF-α expression by increasing methylation of the TNF-α promoter, thereby reducing TNF-α autocrine and inhibiting alveolar macrophage necroptosis [113]. This study reveals a new mechanism of apigenin in inhibiting alveolar macrophage necroptosis, potentially significant for the development of anti-Mycoplasma drugs and providing reference value for future studies on the role of apigenin in other necroptosis-induced inflammatory diseases. Despite apigenin’s many positive effects and lower intrinsic toxicity to normal and cancer cells compared to other structurally related flavonoid compounds, the low solubility in water (1.35 μg/mL) and high permeability may limit its in vivo usage [167,168]. Various delivery systems have been reported to enhance the solubility of apigenin, including liposomes, polymeric micelles, and nano-suspensions [169,170,171,172]. On the other hand, apigenin’s poor bioavailability, its transformation into larger molecules in tissues, and susceptibility to methylation, sulfation, and glucuronidation, metabolized through UDP-glucuronosyltransferase (UGT) more rapidly in the intestines than in the liver, affect its biological distribution and activity [173,174,175]. The injectable nanomedicine delivery system developed by Karim et al., showing good encapsulation efficiency and drug loading, with low complement consumption and non-toxicity to cells, suggests that this system could be an effective method to improve the bioavailability of apigenin [176].

Acteoside (AC), also known as verbascoside, a phenylpropanoid glycoside isolated from various dicotyledonous plants, exhibits a wide range of pharmacological activities, including anti-inflammatory, antioxidant, antiviral, anticancer, and neuroprotective properties [177,178]. Studies have found that AC therapy improves lipopolysaccharide-induced ALI by inhibiting NF-κB mediated inflammatory events [179]. A recent study reported for the first time that AC can protect against RSV-induced pulmonary inflammation and necroptosis in vitro and in vivo [180]. AC treatment downregulates the expression of high mobility group box B (HMGB1), RIP1, RIP3, and MLKL. Additionally, AC exhibits anti-inflammatory effects by reducing levels of TNF-α, IL-1β, and IL-6 in lung tissue and bronchoalveolar lavage fluid (BALF) induced by RSV [180]. The beneficial role of AC in inhibiting necroptosis suggests that it could be a potential new candidate drug for treating RSV infections [180]. Although AC has been subjected to multiple clinical trials without reports of severe adverse reactions, its bioavailability is relatively low [181]. Intravenous administration of AC in rats shows a bioavailability of only 0.12%, and its absolute bioavailability in beagles is about 4% [182,183]. The low bioavailability of AC may be related to its poor absorption rate and bioaccessibility, but there is potential to enhance AC’s bioavailability through liposomal and chitosan encapsulation. Chitosan-coated AC liposomes (CS-AC-Lip) have shown improved pharmacokinetic parameters [182,183,184,185].

Crocetin, one of the active components found in the fruit of Gardenia, is known for its cardioprotective, hepatoprotective, neuroprotective, antidepressant, and antiviral properties [186,187,188]. Receptors such as TNFR1, TRAIL-R1, and TNFRSF10B, when bound to members of the TNF superfamily, initiate necroptosis [189,190,191,192]. Studies have found that crocetin inhibits necroptosis by downregulating the Tnfrsf10b gene at the transcriptional level, providing good protection in a mouse model of radiation-induced lung injury [193]. In a study by Almodóvar et al., the maximal concentration of crocetin in the blood of 13 healthy human volunteers was detectable approximately 60–90 min after the ingestion of different concentrations of Gardenia extract, showing a dose-dependent pharmacokinetic response [194]. In multiple clinical studies involving healthy volunteers, no significant adverse reactions were observed following oral administration of crocetin [195,196,197]. While its low water solubility, poor oral absorption, and low bioavailability have hindered therapeutic applications, advancements in derivatization and formulation preparation technologies are expanding the potential applications of crocetin [198,199].

Aloperine (Alo), a quinolizidine alkaloid extracted from Sophora alopecuroide, possesses potent antioxidative and anti-inflammatory abilities [200]. Studies have found that aloperine can counteract LPS-induced macrophage activation by reducing Toll-like receptor 4 (TLR4) and myeloid differentiation factor (Myd-88) levels and preventing nuclear translocation of nuclear factor-κB (NF-κB) [201]. Cui and colleagues found that Alo significantly alleviates tissue pathological lung injury in LPS-induced ALI mice, reducing total protein and neutrophil recruitment in bronchoalveolar lavage fluid and improving lung necroptosis in mice [202]. LPS stimulates TLR4 activation in the presence of caspase inhibition, with active TLR4 promoting the formation of necrosomes composed of TRIF, RIPK3, and MLKL, thereby inducing necroptosis [48,49]. Alo blocks the TRIF/RIPK3/MLKL signaling pathway, significantly reducing LPS-induced alveolar epithelial cell necroptosis [202]. However, aloperine’s clinical application is constrained by certain limitations. A key concern is its toxicity. A study indicated that intraperitoneal administration of aloperine at a dose of 16 mg/kg could cause reversible damage to the liver and kidneys in mice [203]. Increased dosages to compensate for low bioavailability may also augment toxicity to bodily systems. The in vivo pharmacokinetic properties of aloperine are yet to be fully established. Aloperine’s poor solubility in aqueous solutions indicates that specific drug delivery systems might be alternative options to overcome these barriers and enhance target efficacy [204,205].

### 3.3. Other Interventions

STING, also known as TMEM173, ERIS, MITA, or MPYS, acts as a cytosolic sensor for cyclic dinucleotides and adaptor protein for various intracellular DNA sensors, including IFI204, cGAS, ZBP1, and DDX41, playing a critical role in innate immunity [206,207]. STING has been implicated in various bacterial infections and elicits different immune responses depending on the pathogen and infection model. Research has shown that STING can prevent lung Staphylococcus aureus infection by inhibiting necroptosis, with STING deficiency leading to increased mouse mortality, higher BALF and lung bacterial burden, severe lung structure damage, increased inflammatory cell infiltration, and cytokine secretion [208].

Melatonin, besides regulating the sleep-wake cycle, has shown beneficial effects in various disease models, such as antioxidative and anti-inflammatory properties [209]. Its membrane receptors include high-affinity melatonin receptors 1 (MT1), MT2, and MT3. Activation of MT1/MT2 in animal models has been shown to prevent acute lung injury and rat lung damage caused by polluted air [210,211]. Melatonin has been found to inhibit LPS-induced bronchial epithelial cell necroptosis and counteract chronic lung inflammation caused by LPS by targeting membrane receptors MT1/MT2 [212]. Although the detailed mechanisms of how melatonin’s MT1/MT2 inhibit the necroptosis pathway are not fully explored, metabolomic analysis suggests that melatonin may reduce necroptosis induced by LPS-induced metabolic disturbances, such as alterations in the alanine, aspartate, and glutamate metabolic pathways [212,213]. For instance, key metabolites such as L-alanine and L-asparagine significantly decrease under melatonin treatment, indicating that inhibited alanine and glutamate metabolism under melatonin might reduce necroptosis. Furthermore, the consumption of pyruvate and inhibition of mitochondrial pyruvate carrier have been demonstrated to suppress TNF-mediated necroptosis [212,213]. These findings highlight melatonin’s potential role in reducing necroptotic cell death in pneumonia and suggest the need for further investigation into its specific mechanisms of action.

Selenium (Se), an essential trace element for healthy organisms, functions through selenoproteins, most of which are enzymes involved in redox reactions [214]. Selenium deficiency-induced oxidative stress has been found to cause lung inflammation, cell apoptosis, and necroptosis in calves [215]. Selenomethionine (SeMet) has been shown to reduce oxidative damage, diminish (NLRP3) inflammasome activation, and decrease chicken lung necroptosis induced by LPS [216]. Mechanistic studies indicate that SeMet reduces LPS-induced necroptosis by increasing miR-15a expression, inhibiting oxidative stress, and reducing LPS-induced upregulation of JNK, thereby decreasing NLRP3 inflammasome-induced lung necroptosis [216].

## 4. Future Perspectives and Conclusions

Currently, severe pulmonary inflammation caused by a variety of pathogens remains a significant cause of global mortality. The emergence of COVID-19 in 2019 and the subsequent pandemic have led to an estimated 4 million deaths worldwide, triggering extensive research into adjunctive therapies. Antibiotics are the mainstay of pneumonia treatment; however, optimizing antibiotic therapy, considering the physiological changes and disease severity related to severe lung infections, presents challenges [217]. Patients infected with COVID-19 often suffer from secondary infections with pathogens such as Staphylococcus aureus, further increasing morbidity and mortality rates [218]. Recent occurrences of co-infections with Mycoplasma pneumoniae and influenza viruses in various parts of China have led to worsened patient outcomes and increased complications [219,220,221].

These pathogens activate various signaling pathways in the lungs, inducing necroptotic cell death. Emerging evidence suggests that necroptosis plays a critical role in the development of pneumonia. Necroptosis can be triggered by multiple stimuli and proceeds via different pathways, with key roles played by RIPK1, RIPK3, and MLKL. This process results in membrane permeabilization, cytokine release, tissue damage, and immune dysregulation. Given the role of necroptosis in the pathogenesis of pneumonia, the use of inhibitors targeting necroptotic pathways as adjunctive drugs is proposed to enhance treatment outcomes, particularly in severe pneumonia caused by mixed infections.

Substantial evidence indicates that the synergistic action between existing antibiotics and bioactive compounds is a unique and promising concept [222,223,224]. A study found that oleanolic acid, when used in conjunction with erythromycin, gentamicin, ciprofloxacin, and norfloxacin, exhibited synergistic effects against Gram-negative bacterial strains, potentially aiding in antimicrobial therapy [225]. This review has discussed small molecule inhibitors targeting key components of the necroptotic pathway, including RIPK1 and RIPK3. These inhibitors can prevent the formation of necrosomes and subsequent necroptotic cell death. Additionally, other drugs such as antioxidants and immunomodulatory compounds have shown potential in alleviating tissue damage and inflammation induced by necroptosis. Notably, most research on necroptosis inhibitors has been conducted in vitro, necessitating further evaluation for clinical trial feasibility and in vivo use.

In summary, targeting necroptosis represents a significant advancement in the development of new therapeutic strategies for pneumonia. Especially in the context of antibiotic resistance, the combination of necroptosis inhibitors with existing antimicrobial therapies is a promising strategy for future pneumonia treatment development.

## Data Availability

No new data were created.
